# Corded and hyalinized endometrioid carcinoma: a case report and literature review

**DOI:** 10.3389/fonc.2025.1677355

**Published:** 2026-01-12

**Authors:** Rong Liu, Yani Wang, Weichen Shi, Yanan Wang, Jiangong Hu

**Affiliations:** 1 Department of Pathology, The Second Affiliated Hospital of Tianjin University of Traditional Chinese Medicine, Tianjin, China; 2 Department of Gynecology, The Second Affiliated Hospital of Tianjin University of Traditional Chinese Medicine, Tianjin, China

**Keywords:** corded and hyalinized, CTNNB1, endometrioid carcinoma, molecular classification, β-catenin

## Abstract

Corded and hyalinized endometrioid carcinoma (CHEC) is a rare morphological subtype of endometrioid carcinoma (EC), characterized by a biphasic pattern consisting of epithelial or spindled tumor cells embedded within hyalinized stroma, merging with conventional low-grade EC. We present a case of CHEC in a 24-year-old woman who presented with irregular vaginal bleeding. Imaging showed a mass protruding toward the cervix, and subsequent tumor resection confirmed the diagnosis of CHEC. Immunohistochemical staining for β-catenin and CTNNB1 gene mutation analysis were helpful in the diagnosis of CHEC, and molecular subtyping provided valuable prognostic information to a certain extent. Postoperatively, the patient received progestin therapy for 6 months and remained recurrence-free. Given the rarity of CHEC, distinguishing this disease from other biphasic uterine tumors is crucial. This report aims to enhance recognition of this rare entity, facilitating its accurate diagnosis and preventing inappropriate therapeutic interventions.

## 1 Introduction

Endometrioid carcinoma with cord-like component and hyalinized stroma is a rare histological variant of EC. It was first reported by Murray et al. in 2005 (1), who described the clinicopathological features and proposed the designation of “corded and hyalinized endometrioid carcinoma” for this variation. CHEC exhibits a biphasic appearance, the typical low-grade EC merging with corded and hyalinized component without boundaries (1–7). There is no particularity for its clinical symptoms and imaging manifestations. For pathologists, it is vital to recognize its biphasic morphology and differentiate it from other uterine tumors with similar characteristics, especially carcinosarcoma and dedifferentiated endometrial carcinoma due to their high invasiveness. We provide a case of CHEC, meanwhile, review the available literature to enhance the understanding of this disease.

## 2 Case presentation

**Figure 1:**
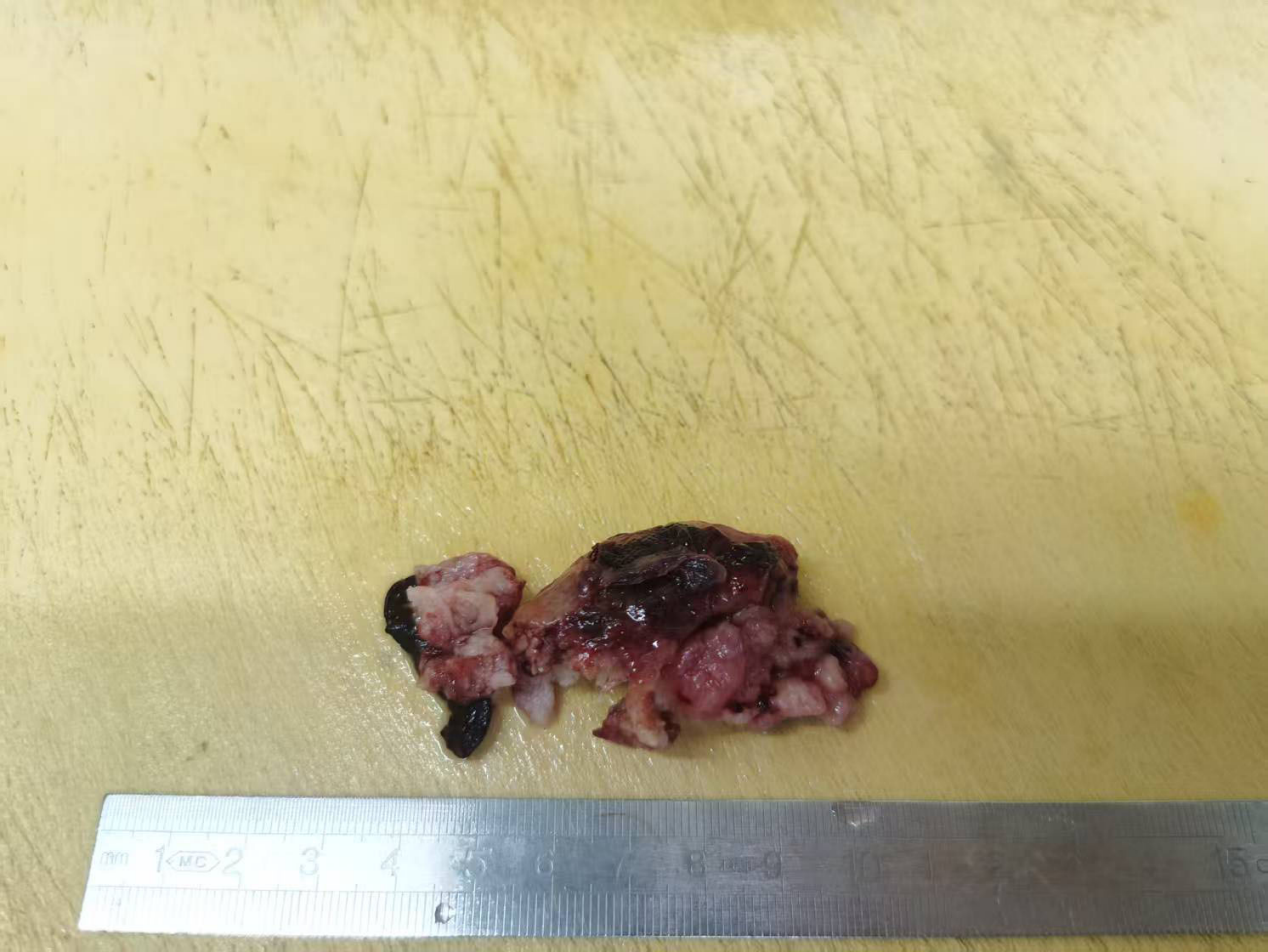
Grossly, the fragmented tumor was grayish red in color and friable in texture, measuring 5x4.8x1.7 cm, with some areas presenting a nodular appearance.

**Figure 2:**
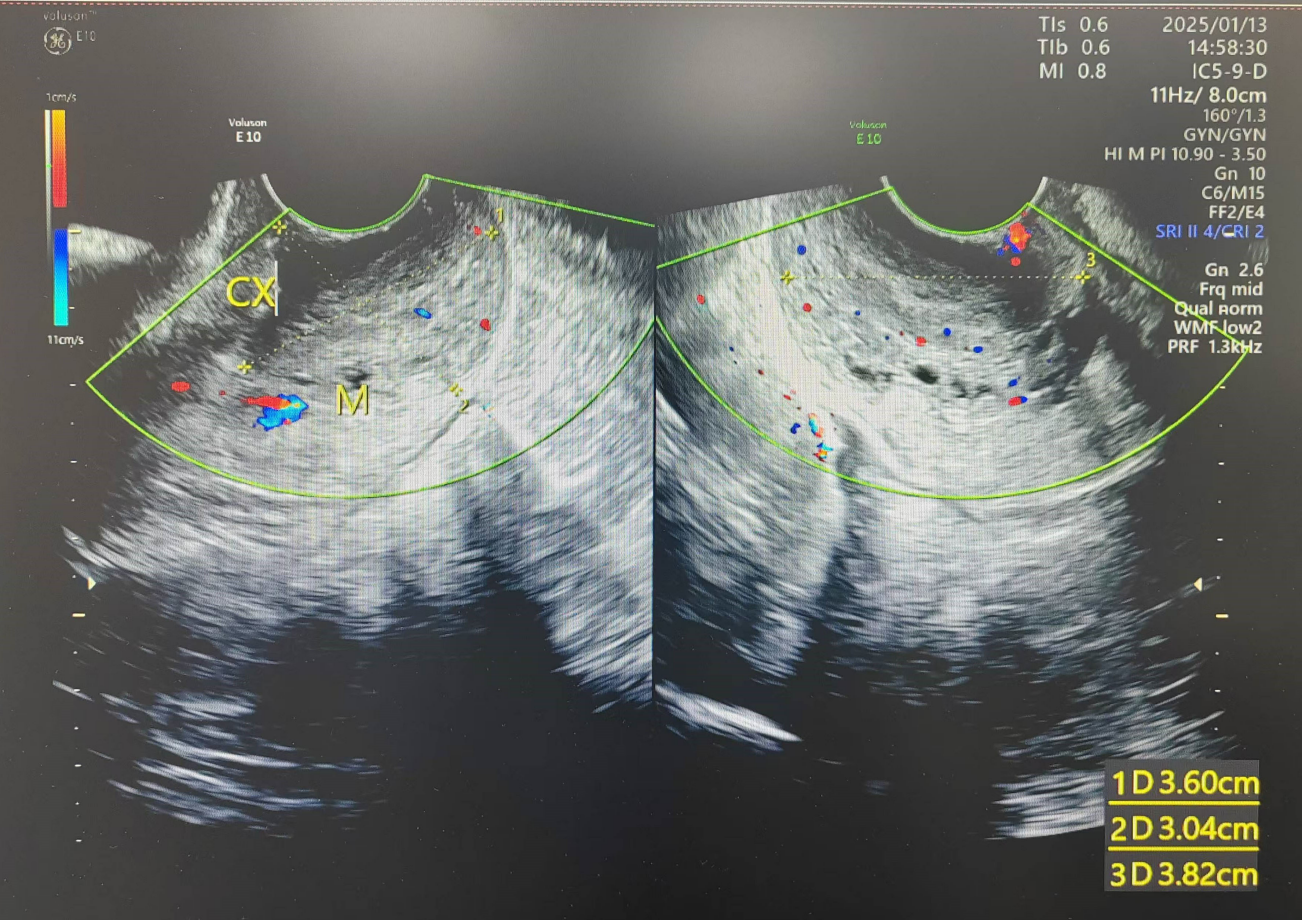
Ultrasonography demonstrated an area of heterogeneous echoes at the cervix with unclear boundaries, measuring 3.8×3.6×3.0 cm.

A 24-year-old woman was admitted to the Department of Gynecology of our hospital in January 2025. The patient presented with irregular vaginal bleeding of unknown etiology persisting for over 3 months, accompanied by dizziness, headache, fatigue and severe anemia. She had a regular menstrual cycle, G0P0, and her last menstrual period was at the end of September 2024. She denied any family history of genetic disorders or tumors. Gynecological examination revealed a mass protruding from the uterine isthmus through the external cervical os to the vagina, measuring 7x4 cm, with a fragile texture (**Figure 1**). Ultrasonography demonstrated an area of heterogeneous echoes in the cervix with unclear boundaries, measuring 3.8×3.6×3.0 cm, with visible blood flow signals (**Figure 2**). The uterus was of normal morphology. The endometrium was 1.2 cm thick, and the echo within it was uneven. Bilateral ovaries exhibited polycystic alterations. MRI revealed a soft tissue signal mass in the cervical region, while the endometrial mucosa and myometrium of the uterine fundus and body appeared unremarkable without significant abnormalities. The serological level of AFP, CA125, CA199, CEA, hCG, HE4, and SCC were all normal. Preoperative evaluation showed a positive HPV16 result and a normal liquid-based cytology report. The patient underwent tumor resection and endometrial curettage concurrently on January 20, 2025. Following the insertion of the hysteroscope under intravenous general anesthesia, a mass was observed prolapsed into the cervical canal, with its base sessile at the uterine isthmus. The mass was completely resected at its base using a plasma resectoscope loop. Subsequently, endometrial curettage was performed, yielding copious tissue. Both the resected mass and the curettage specimens were submitted together for pathological examination.

**Figure 3:**
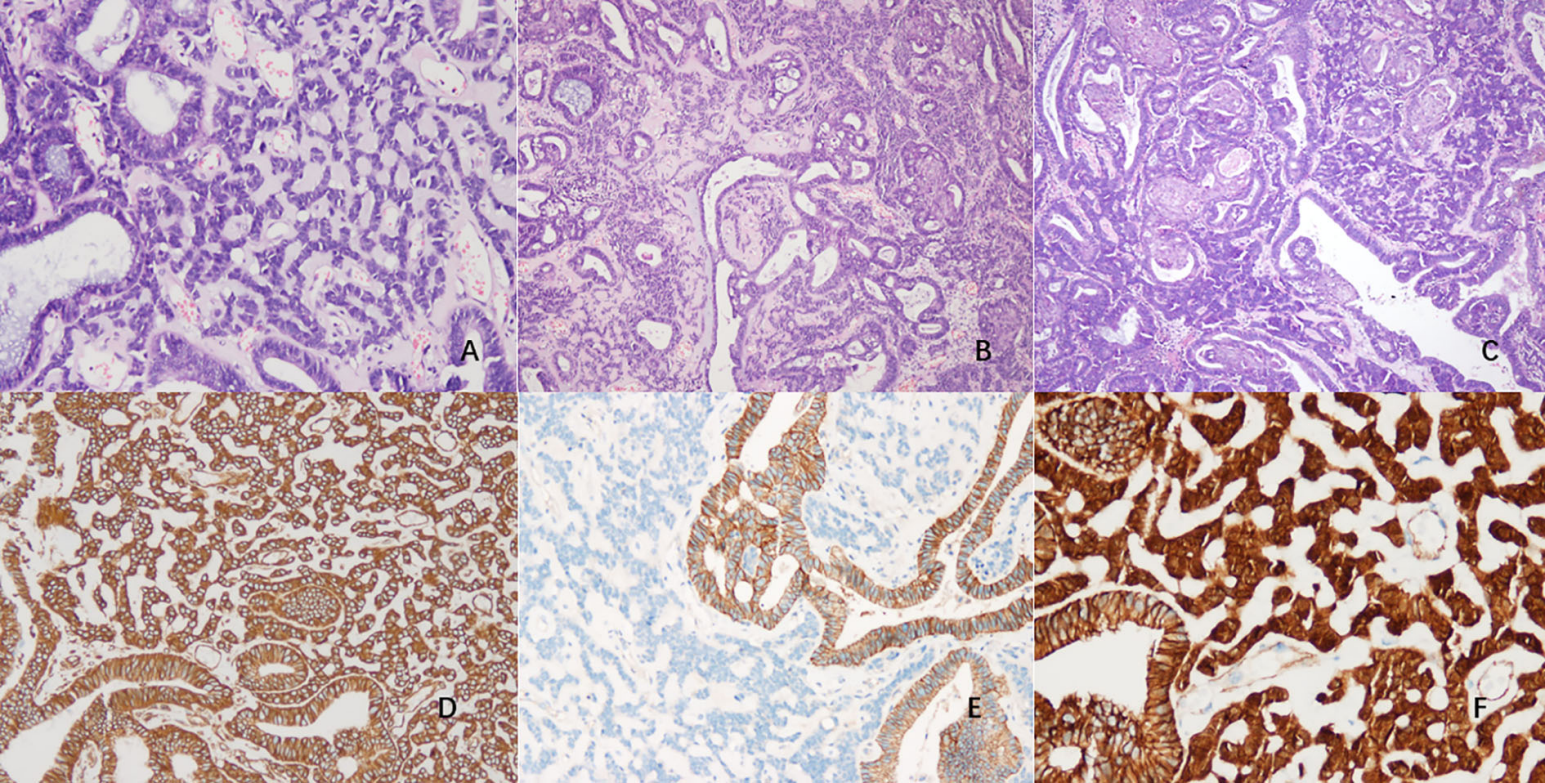
Light microscopic appearance of CHEC **(A–F)**. **(A)** The cords and small clusters embedded within hyalinized stroma, the cells exhibited mild to moderate cytological atypia. (H&E, x100) **(B)** The low-grade EC merging with corded and hyalinized component, forming a biphasic pattern. (H&E, x40) **(C)** Squamous metaplasia nests located within both areas. (H&E, x40) **(D)** Immunohistochemical staining showed diffuse positive for Vimentin. (x100) **(E)** The corded and hyalinized regions were negative for E-cad. (x100) **(F)** β-catenin showed positive in both nucleus and cytoplasm, while the adjacent glandular epithelium demonstrated membranous expression. (x200).

Microscopically, the glandular component was consistent with low - grade EC in a background of complex atypical endometrial hyperplasia in the resected tumor specimen. The stroma was hyalinized, within which, epithelioid and spindled tumor cells were arranged in cords and small clusters (**Figure 3A**). Cellular cords interwove with each other to form a network. EC and corded component were closely integrated, presenting a distinct biphasic pattern in the tumor (**Figure 3B**). The cells in corded area exhibited mild or moderate cytological atypia and had lower mitotic activity. The corded and hyalinized component accounted for approximately 70% of the tumor. Squamous metaplasia nests were observed in both components, and the majority of them were located within the EC area (**Figure 3C**). Similar histological features can also be seen in endometrial resection specimens, the percentage of corded and hyalinized component was about 50%.

In the EC component, immunohistochemistry revealed strong staining for ER, PR, and Vimentin, with β-catenin expression on the membrane. In contrast, PTEN displayed a loss of expression. The Ki-67 index was approximate to 25%. In the corded and hyalinized regions, it was diffusely positive for Vimentin (**Figure 3D**), but was negative for ER, PR, CKpan, E-cad (**Figure 3E**), CD10 and WT-1. Sex-cord markers such as α-inhibin and CR were also negative. β-catenin showed positivity in both nucleus and cytoplasm (**Figure 3F**). The Ki-67 index was around 15%. In both two components, P53 exhibited wild-type expression, and P16 displayed patchy positive. No loss of expression of MLH1, MSH2, MSH6 or PMS2 was detected. The squamous metaplasia areas were positive for CK5/6 and focally positive for P63 and P16, and the Ki-67 index was less than 5%.

Next-generation sequencing (NGS) identified two somatic mutations: p.S37C (c.110C>G) in CTNNB1 exon 3 and p.D24G (c.71A>G) in PTEN exon 1. No pathogenic variants were detected in the exonuclease domain of POLE and TP53 gene. Microsatellite instability (MSI) testing exhibited MSI-L/MSS status. According to The Cancer Genome Atlas (TCGA) molecular classification system, this case fell within the No Specific Molecular Profile (NSMP) group for endometrial carcinoma.

Final pathological diagnosis confirmed CHEC, FIGO grade 1. According to the *Chinese Society of Clinical Oncology (CSCO) Guidelines for Diagnosis and Treatment of Endometrial Carcinoma (2025)*, continuous oral megestrol acetate at 160–320 mg/day is recommended as a Category I treatment option for fertility-preserving management of endometrial carcinoma. Postoperatively, the patient received oral megestrol acetate 160 mg twice daily for 6 months. During treatment, she underwent transvaginal color doppler ultrasound and hysteroscopy with endometrial biopsy every 3 months. In April and July 2025, pathological evaluations of endometrial biopsies from two separate hysteroscopies both indicated complete response (CR). Oral medication was subsequently discontinued, and a Mirena (levonorgestrel-releasing intrauterine system, LNG-IUS) was placed for maintenance therapy. Follow-up was planned every 3–6 months until the patient plans to conceive or until disease progression. The patient has remained recurrence-free for 8 months after first CR.

## 3 Discussion

### 3.1 Clinical features

As of June 2025, 7 series studies encompassing 68 CHEC patients were identified. The clinicopathological features were summarized in **Table 1** (1–7). The age range was 19-83 years, with a mean age of 49 years —14 years younger than that of classic EC (63 years) (8). Approximately 56% of cases occurred in patients under 50 years of age. Its clinical presentation was abnormal vaginal bleeding or menorrhagia, while a minority of patients were asymptomatic. Several established risk factors for EC were observed in CHEC patients, including obesity, insulin resistance, infertility, anovulation, and polycystic ovary syndrome (PCOS) (1, 6). Tumor dimensions varied considerably from microscopic foci to large lesions measuring up to 15 cm in diameter. Larger tumors usually presented as polypoid masses with grayish-white in color. Cases involving the lower uterine segment demonstrated cervical stromal invasion in some instances (4). Of the 55 CHEC patients with FIGO staging data, 39 (70.9%) were classified as Stage I, 8 (14.5%) as Stage II, and 7 (12.7%) as Stage III. The majority of the patients were at an early stage, and only 1 case (1.8%) progressed to Stage IV due to confirmed bone metastasis. The present case involved a young, nulliparous patient with obesity (BMI 33.2) and imaging findings suggestive of PCOS. The tumor was notably large, exhibiting a polypoid morphology with protrusion through the cervix. Due to the absence of hysterectomy, assessment of cervical stromal invasion and FIGO staging were not feasible.

**Table 1:** Summary of clinicopathological characteristics of 7 series studies.

	Murray SK, 2005 (1)	Wani Y, 2009 (2)	Sun YH, 2016 (3)	Ladwig NR, 2020 (4)	Safdar NS, 2021 (5)	Travaglino A, 2022 (6)	Pors J, 2023 (7)	Summary
Sample size (n)	31	6	5	7	7	6	6	68
mean age, years (range)	52 (25-83)	46 (38-57)	33 (29-39)	48.4 (19-69)	48.9 (34-68)	57.5 (29-74)	40.3 (23-58)	49 (19-83)
<30	2	0	1	1	0	1	2	7 (10.3%)
30-50	15	4	4	3	5	0	2	31 (45.6%)
50-70	8	2	0	3	2	3	2	22 (32.4%)
>70	6	0	0	0	0	2	0	8 (11.8%)
FIGO Stage (n)								
- I	20	NA	3	4	2	5	5	39/55 (70.9%)
- II	5	NA	0	2	1	0	0	8/55 (14.5%)
- III	1	NA	1	1	2	1	1	7/55 (12.7%)
- IV	1	NA	0	0	0	0	0	1/55 (1.8%)
Grade (n)								
-low-grade	31	NA	5	6	6	0	6	54/62 (87.1%)
-high-grade	0	NA	0	1	1	6	0	8/62 (12.9%)
% of the corded component	10-90	<10- >30	10-60	<10- >90	15-80	15-50	<5-20	<5- >90
squamous metaplasia	22/31	5/6	3/5	7/7	7/7	6/6	3/6	53/68 (77.9%)
osseous/chondroid metaplasia	8/31	NA	0/5	1/7	0/7	3/6	2/6	14/62 (22.6%)
LVSI	7/27	NA	0/5	3/7	1/5	0/6	NA	11/50 (22%)
Nuclear β-catenin	NA	6/6	NA	7/7	6/6 (1 high-grade NA)	2/6 (diffuse)	6/6	27/31 (87.1%)
P53 overexpression	1/10	1/6	0/5	1/7	1/7	2/6	0/6	6/47 (12.8%)
MMR loss	NA	NA	NA	1/7	0/7	3/6	NA	4/20 (20%)
CTNNB1 mutation	NA	4/6	NA	7/7	NA	0/6	NA	11/19 (57.9%)
molecular classification								
-POLE mutant	NA	NA	NA	0/7	0/7	0/6	NA	NA
-MMRd	NA	NA	NA	1/7	0/7	3/6	NA	NA
-p53-abnormal	NA	NA	NA	1/7	1/7	2/6	NA	NA
-NSMP	NA	NA	NA	5/7	6/7	1/6	2/6	14/26 (53.8%)
Progesterone therapy	0/30	NA	0/5	1/7	1/7	NA	4/6	6/55 (10.9%)
tumor resection/ D&C	3/30	NA	1/5	0/7	2/7	0/6	0/6	6/61 (9.8%)
hysterectomy	27/30 (2 with RAD)	NA	4/5 (1 with Adjuvant therapy)	7/7 (4 with Adjuvant therapy)	5/7 (3 with Adjuvant therapy)	6/6	6/6	55/61 (90.2%)
Follow up								
-NED	NA	NA	4/4	2/6	2/3	5/5	5/6	33/42 (78.6%)
-AWD	1/18	NA	0/4	2/6 (1 with lung metastases)	0/3	0/5	1/6 (recurrence in Vaginal cuff)	4/42 (9.5%)
-DOD	1/18	NA	0/4	2/6 (1 had a lung mass)	0/3	0/5	0/6	3/42 (7.1%)
-DOC	1/18	NA	0/4	0/6	1/3	0/5	0/6	2/42 (4.8%)

RAD, radiation; D&C, dilation and curettage; NED, no evidence of disease; AWD, alive with disease; DOD, died of disease; DOC, death from other causes; NA, not available.

### 3.2 Pathological findings

Histologically, CHEC is characterized by an intimate admixture of classic low-grade EC and corded and hyalinized components without clear demarcation. The cord-like element typically appears around the EC glands, with epithelioid or spindled/fusiform cells arranged in short cords, small clusters, or as individual cells embedded in hyalinized stroma, resembling mesenchymal element and creating a biphasic pattern. A subset of cases exhibits less prominent stromal hyalinization, with corded cells arranged in solid sheets (1). In fact, the corded and hyalinized features may also occur in endometrial hyperplasia with or without cytologic atypia, or in hyperplasia with architectural complexity bordering on well-differentiated EC (7). Similar findings were consistent with our case. The proportion of corded and hyalinized component varies significantly among reported cases, ranging from <5% to >90%. Furthermore, these areas are typically located superficially, some cases demonstrate deep tumor involvement or intricate intermingling with the carcinoma component (4, 6). When myometrial invasion occurs, it usually involves the carcinoma component, though rare cases show invasion by both elements (1, 5). In our case, the corded and hyalinized component exceeded 50% in both resection and curettage specimens, showing extensive intermingling with carcinoma rather than superficial restriction. Squamous differentiation, a diagnostic hallmark of CHEC, was present in 77.9% of cases and could be observed in both patterns. Additionally, 22.6% of cases showed osseous or chondroid metaplasia, and lymphovascular space invasion (LVSI) was observed in 22% of cases. The current case demonstrated extensive squamous differentiation, predominantly localized to the carcinoma area and rarely occurred in corded and hyalinized component. Meanwhile, neither chondroid nor osseous metaplasia was identified.

The EC component typically shows diffuse expression of epithelial markers including CKpan and EMA, with variable positive for ER, PR, and PAX-8. In contrast, the corded cells show markedly reduced or absent expression of these markers. Vimentin is positive in both components, but its staining appears more diffuse within the corded areas. It is negative for myogenic markers such as SMA and Desmin as well as sex-cord markers including α-inhibin, Calretinin, CD99, and WT-1. E-cadherin expression is completely absent in the corded and hyalinized area, while the EC component retains complete membranous positive. Diffuse nuclear β-catenin positivity is consistently observed in the corded component of the vast majority of cases (1–7). Multiple studies have shown that β-catenin plays a significant role in the formation of CHEC morphology by participating in epithelial-mesenchymal transition (EMT) (2, 9, 10), and the activation of the Wnt/β-catenin pathway leads to the aberrant activation of EMT-related genes including SNAIL and ZEB1 (11, 12). Specifically, it is diffuse and intense for β-catenin staining in the corded and hyalinized areas, while the adjacent EC cells show relatively weak staining. EC glands far from these areas rarely or never show nuclear staining. This suggests that the Wnt signaling pathway is most active in the transitional zone between the glandular and the corded and hyalinized components (4).

The majority of CHEC are low-grade (54/62, 87.1%), and the cells in the corded and hyalinized component typically exhibit mild-to-moderate atypia, with a mitotic index generally lower than that of the EC. However, 8 cases demonstrated either focal or diffuse high-grade EC, accompanied by corded cells showing widespread high-grade atypia and significantly increased mitotic activity (5-45/10 HPF). Of these, only 3 showed characteristic diffuse nuclear β-catenin staining, while the remaining cases were focal positive or negative (4–6). Additionally, P53 overexpression and dMMR predominantly associated with the high-grade morphology of CHEC, suggesting that these two abnormalities might be the basis for the development of high-grade atypia (13). In our case, no abnormal expression of P53 and MMR protein was observed, and the immunohistochemical profiles was consistent with conventional CHEC described in the literature.

### 3.3 Molecular analysis

Comprehensive literature analysis demonstrated the mutation rate of CTNNB1 in CHEC was 57.9% (11/19), with most mutations occurred in low-grade lesions. The close relation between CTNNB1 exon 3 mutations and nuclear β-catenin accumulation has led to the adoption of β-catenin immunohistochemistry as a reliable surrogate marker for CTNNB1 mutational status (14, 15). However, this pattern differs in high-grade CHEC. The study by Travaglino et al. revealed that although high-grade CHEC showed varying degrees of β-catenin expression, no CTNNB1 mutations were found in all cases, suggesting the possibility of the tumors harbored mutations outside the hotspots in exon 3 or other alterations in the Wnt/β-catenin pathway (6). Ladwig et al. (4) reported a case of high-grade CHEC harboring concurrent TP53 and RB1 mutations. According to TCGA classification, the majority of CHEC (53.8%) were classified as NSMP subtype, similar to classic low-grade EC. Among 8 high-grade CHEC cases, 7 exhibited MMR-deficiency or p53-abnormality. However, two additional high-grade CHEC cases reported in 2025 both fell into the NSMP group, confirming the molecular heterogeneity of high-grade CHEC (16). Our case showed molecular features of low-grade CHEC (CTNNB1 and PTEN mutations) and were classified as NSMP.

### 3.4 Treatment and prognosis

Among the 61 patients, 55 underwent hysterectomy, most with concurrent bilateral adnexectomy or pelvic lymphadenectomy, 6 patients received only endometrial curettage and tumor resection. Approximately 85% of cases were at stage I or II, and 88.1% of patients achieved either disease-free survival or alive with disease, indicating that most CHEC have an early clinical stage and a favorable outcome. However, Ladwig’s study reported poor outcomes in 4 NSMP-classified cases, suggesting their aggressive clinical course might associate with concurrent CTNNB1 mutations in NSMP tumors (4). While existing studies indicated CTNNB1 mutations alone cannot drive carcinogenesis (17, 18), due to the molecular heterogeneity within NSMP group, CTNNB1-mutated EC demonstrate higher recurrence rate than wild-type counterparts (14, 19, 20). Notably, 3 of these 4 cases exhibited cervical stromal invasion and lymphovascular space invasion, prompting the authors to recommend comprehensive prognostic evaluation incorporating these recurrence-related pathological features. Other studies failed to demonstrate significant correlations between poor outcome and MMR-deficient/p53-abnormal molecular subtypes, though larger cohort validation remains warranted. Due to the patient’s young age and nulliparous status in our case, oral progestin therapy followed by subsequent LNG-IUS treatment was initiated after tumor resection. With continued follow-up, no tumor recurrence has been detected to date.

### 3.5 Differential diagnosis

Given the biphasic pattern of CHEC, differential diagnosis should include various uterine biphasic tumors and neoplasms with sex cord-like differentiation. Carcinosarcoma is an aggressive biphasic tumor, usually demonstrates sharply demarcated high-grade carcinoma and sarcoma components, potentially with heterologous elements such as rhabdomyosarcoma or chondrosarcoma. CHEC shows ossification and chondrogenesis sometimes, but it rarely contains high-grade heterologous components. Occasionally, there are morphologically distinctive high-grade CHECs, which are almost entirely composed of corded and hyalinized component, with obvious cellular atypia, posing a significant challenge for pathological diagnosis, especially in biopsy specimens. The frequent presence of squamous morules and diffuse nuclear β-catenin staining strongly supports CHEC diagnosis. Furthermore, most carcinosarcomas harbor TP53 mutations rather than CTNNB1 (4). Dedifferentiated endometrial carcinoma is a biphasic neoplasm composed of both differentiated and undifferentiated tumor components. The differentiated component typically consists of well-defined EC, while the undifferentiated component is characterized by small round cells with loss of cellular adhesion (21). Contrasting with CHEC, it lacks cord-like structures and stromal hyalinization, with a sharp demarcation between two components. Molecular genetics reveal that most dedifferentiated carcinomas have abnormal MMR protein expression, and some of these cases belong to the tumor spectrum of Lynch syndrome (22).

Although rare, sex cord-like structures can occur in various neoplastic and non-neoplastic uterine lesions. Uterine tumors resembling ovarian sex cord tumors (UTROSCT), endometrial stromal nodules, and low-grade endometrial stromal sarcomas may all demonstrate extensive sex cord-like differentiation, manifesting as anastomosing cords, trabeculae, nests, islands, tubules, or diffuse patterns. However, they lack both EC component and hyalinized stroma. It was positive for true sex cord markers but negative for EMA (23). When sex cord-like areas are present in curettage specimens, it can be difficult to distinguish CHEC from epithelioid leiomyomas with nested, cord-like, or trabecular structures and Sertoli-like EC (24). The former are positive for myogenic markers, while the latter variably express sex-cord markers and behave as high-grade EC.

## 4 Conclusion

In summary, based on the TCGA molecular classification framework, our case provided a detailed description of the pathological morphology, immunophenotype, and molecular profile of a young patient with early-stage CHEC and documented the achievement of pathological CR following fertility-preserving treatment. However, as a single-case report, the pathological features observed may not fully represent the complete morphological spectrum and molecular heterogeneity of CHEC. Additionally, the relatively short follow-up period limits the assessment of long-term outcomes. Future research should rely on accumulated pathological data from larger case series and long-term follow-up to further explore the clinicopathological correlations and prognostic factors of CHEC.

## Data Availability

The original contributions presented in the study are included in the article/supplementary material. Further inquiries can be directed to the corresponding author.
